# A *klotho* gene single nucleotide polymorphism is associated with the onset of stroke and plasma klotho concentration

**DOI:** 10.18632/aging.101728

**Published:** 2018-12-31

**Authors:** Serina Yokoyama, Ryosuke Oguro, Koichi Yamamoto, Hiroshi Akasaka, Norihisa Ito, Tatsuo Kawai, Hiroshi Kusunoki, Yasushi Takeya, Miyuki Takeya-Onishi, Hiroko Yamamoto-Hanasaki, Ken Sugimoto, Kazunori Ikebe, Yasuyuki Gondo, Mitsuru Ohishi, Kei Kamide, Hiromi Rakugi

**Affiliations:** 1 Department of Geriatric and General Medicine, Osaka University Graduate School of Medicine, Suita, Osaka 565-0871, Japan; 2 Division of Health Sciences, Osaka University Graduate School of Medicine, Suita, Osaka 565-0871, Japan; 3 Department of Prosthodontics, Gerodontology and Oral Rehabilitation, Osaka University Graduate School of Dentistry, Suita, Osaka 565-0871, Japan; 4 Department of Clinical Thanatology and Geriatric Behavioral Science, Osaka University Graduate School of Human Sciences, Suita, Osaka 565-0871, Japan; 5 Department of Cardiovascular Medicine and Hypertension, Graduate School of Medical and Dental Science, Kagoshima University, Kagoshima, Kagoshima 890-8544, Japan; * Equal contribution

**Keywords:** klotho, stroke, atherosclerosis, SNP, plasma klotho concentration

## Abstract

*Klotho* protects against development of multiple age-related disorders, including cardiovascular diseases. We assessed whether a human *klotho* single nucleotide polymorphism (SNP) rs650439 is associated with the onset of stroke in hypertensive patients and plasma klotho concentration in the general population. Five hundred and twenty-three patients with hypertension were analyzed for both the presence of rs650439 and onset of stroke. We found that hypertensive patients with the TT genotype of rs650439 (n=52) had a higher incidence of stroke than those with AT (n=257) and AA (n=214) genotypes. Multivariate analysis indicated that the TT genotype was the only risk factor associated with increased incidence of stroke. Plasma *klotho* concentrations were measured in a general population (age=70±1 years) to assess the association between rs650439 and plasma *klotho* concentration. A significant trend was observed in the elderly population where plasma klotho concentration decreased as the T alleles in rs650439 increased. Subjects with a TT genotype had lower plasma klotho concentrations than those with AT+AA genotypes. In conclusion, TT genotype of *klotho* SNP (rs650439) is correlated with an increased incidence of stroke in hypertensive patients, and the mechanism underlying this correlation might involve the effect of rs650439 T allele on plasma klotho concentrations.

## Introduction

Stroke is a leading cause of death and disability in developed and developing countries and prevention of stroke is important for promoting good health and longevity. Management of the known risk factors of stroke such as hypertension, diabetes, dyslipidemia, and smoking is an established approach for stroke prevention. Characterization of the genotypes of specific genes associated with the incidence of stroke is a developing means to identify additional risk factors in individuals. Among genotypes that are reported to affect the lifespan of humans [1,2], some are also known to influence the pathogenesis of age-related diseases including cardiovascular diseases such as stroke [3–5]. *Klotho* (*α-klotho*) is such a gene, as indicated in studies of *klotho*-deficient mice that have a shortened lifespan and demonstrate age-related phenotypes such as atherosclerosis, osteoporosis, and emphysema [6]. The human *klotho* gene has more than 80% homology with the mouse gene [7], and polymorphisms in the human *klotho* gene are anticipated to influence the age-related diseases. In fact, previous studies demonstrated correlations between human *klotho* polymorphisms and bone mineral density [8], glucose metabolism [9], cognitive function [10], cardioembolism [11], coronary artery diseases [12], and stroke [13]. The association of *klotho* single nucleotide polymorphisms (SNPs) with carotid artery intima-media thickness (IMT) as an atherosclerotic marker has also been reported [14]. This study showed that a *klotho* SNP rs650439 (A/T) was significantly correlated with carotid artery IMT, an established predictive marker of stroke [15], in hypertensive patients but not in general population. Further, hypertensive patients with a T allele had a higher risk of developing carotid IMT thickening than those with an AA genotype [14].

In the present study, relationships between rs650439 genotypes and the onset of stroke in hypertensive patients was investigated. In addition, the relationship between rs650439 genotype and plasma klotho concentration was examined. To avoid the　confounding influence of age on the circulating klotho concentration that was previously reported [16], klotho concentrations were measured in blood samples from a population of the same age.

## RESULTS

### Study population for the analysis of stroke onset

Of the 813 participants in the Non-Invasive Atherosclerotic Evaluation in Hypertension (NOAH) study [17–19], follow up was possible for 705 subjects (394 males and 311 females). The median follow-up period was 2378 days (interquartile range;1720-2976). Among the latter subjects, 177 individuals with insufficient blood data and 5 individuals with an indeterminate result in genotyping were excluded, leaving a population of 523 patients with successful genotype determination. Genotype distributions and characteristics of each genotype are provided in Table 1. Genotype distribution of rs650439 in the study subjects was similar to the distribution in Asian populations reported in the National Center for Biotechnology Information (NCBI) database (http://www.ncbi.nlm.nih.gov/projects/SNP/snp_ref.cgi?searchType=adhoc_search&type=rs&rs=rs650439) but Hardy-Weinberg equilibrium (p=0.046) was not observed. No significant differences attributable to gender, age, blood pressure, and prevalence of other risk factors were found among subjects with different genotypes (Table 1). While proportion of subjects receiving antihypertensive drugs was comparable among the genotypes, subjects receiving β-blocker with TT genotypes were less frequent than those with AA+AT genotypes (Table 1).

**Table 1:** The characteristics of study subjects in the analysis of stroke.

	All(n=523)		AA(n=214)	AT(n=257)	TT(n=52)	P value*		AA+AT(n=471)	P value†
male, n(%)	296(57)		108(51)	156(61)	32(62)	0.06		264(56)	0.45
age, year	61±12		62±11	60±12	62±12	0.08		61±12	0.42
BMI, kg/m^2^	24.0±3.3		24.0±3.1	24.0±3.4	23.8±3.8	0.91		23.0±3.2	0.67
systolic BP, mmHg	147±22		147±23	146±21	147±24	0.9		147±22	0.97
diastolic BP, mmHg	86±14		85±14	86±14	86±11	0.71		86±14	0.69
diabetes, n(%)	132(25)		57(27)	64(25)	11(21)	0.71		121(26)	0.47
dyslipidemia, n(%)	279(54)		120(56)	126(49)	33(65)	0.08		246(52)	0.09
CKD, n(%)	141(28)		64(31)	65(26)	12(24)	0.43		129(28)	0.49
smoking, n(%)	127(25)		54(26)	62(25)	11(22)	0.86		116(26)	0.59
drinking, n(%)	192(39)		74(36)	98(41)	20(41)	0.63		172(39)	0.76
Treatment of hypertension, n(%)	236(46)		107(50)	109(43)	20(40)	0.23		216(46)	0.4
CCB, n(%)	174(33.6)		72(33.8)	88(34.3)	15(29.4)	0.8		159(34.0)	0.54
ACE inhibitor**, **n(%)	61(11.7)		27(12.6)	29(11.4)	5(9.6)	0.81		56(11.9)	0.82
ARB, n(%)	78(15.0)		42(19.6)	30(11.8)	6(11.5)	0.048*		72(15.4)	0.55
Diuretics, n(%)	34(6.6)		15(7.0)	16(6.3)	3(8.8)	0.93		31(6.7)	1
βblocker, n(%)	50(9.6)		22(10.3)	27(10.6)	1(1.9)	0.14		49(10.4)	0.047†
αblocker, n(%)	10(1.9)		3(1.4)	6(2.4)	1(1.9)	0.76		9(1.9)	1

BMI, body mass index; BP, blood pressure; CKD, chronic kidney disease; CCB, Ca channel blocker; ACE inhibitor, Angiotensin-converting-enzyme inhibitor; ARB, Angiotensin II receptor blocker

*The significance of differences among three genotypes were determined by ANOVA for continuous variables. The significance of differences among three genotypes were determined by chi-square analysis or Fisher’s exact test for categorical variables.

†The significance of difference vs. patients with TT were determined by Student’s t-test for continuous variables. The significance of difference vs. patients with TT were determined by Pearson’s Chi-square analysis or Fisher’s exact test for categorical variables.

### Relationship between rs650439 and the onset of stroke

**Figure 1:**
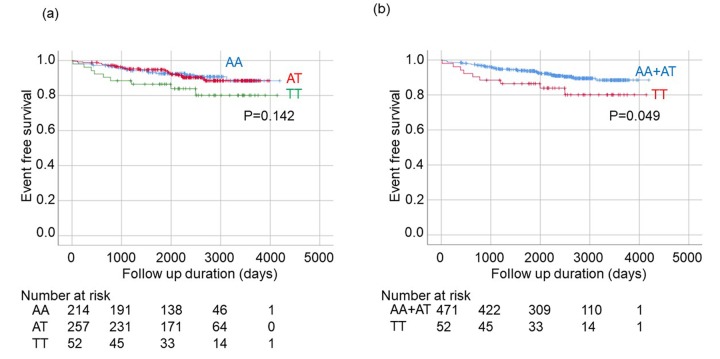
**Kaplan-Meier analysis for onset of stroke among patients with hypertension stratified by rs650439.** (**a**) Comparison among three groups (AA, AT, TT). The significance of differences among three genotypes were determined by Logrank test. (**b**) Comparison between two groups (AA+AT vs. TT). The significance of difference between two groups was determined by Logrank test.

A Kaplan-Meier analysis for onset of stroke among patients with three genotypes (AA vs AT vs TT) suggested that stroke occurred more frequently in subjects with a TT genotype, but differences among groups were not significant (Figure 1a). A similar analysis between two groups (AA+AT vs TT) did indicate a significantly higher incidence of stroke in patients with a TT genotype than in patients with AA or AT genotypes (p=0.049; Logrank) (Figure 1b).

The confounding factors were considered while determining the relationship between rs650439 genotype and the onset of stroke. Using Cox proportional　hazard models, rs650439 genotype (AA +AT or TT) was revealed to be significantly correlated with of the incidence of stroke using the model that included independent variables of age, gender, body mass index (BMI) and treatment of hypertension (Table 2).

**Table 2:** Cox proportional hazard model for the onset of Stroke.

variables	HR	95%CI	p value
rs650429(TT)	2.14	**1.02-4.47**	0.045
age	1.02	1.00-1.05	0.10
gender(male)	1.22	0.68-2.18	0.51
BMI	1.04	0.96-1.13	0.31
Treatment of hypertension	1.48	0.81-2.70	0.20

HR HR, hazard ratio, BMI, body mass index

### Association of rs650439 with plasma klotho protein concentration in 70-year-old community-dwelling elderly subjects

The influence of rs650439 genotype on plasma klotho concentration was examined using data from blood samples from individuals that participated in the SONIC study for which 433 general subjects of 70 ±1 years old were recruited. Genotypes for rs650439 were determined in 417 subjects, and the distribution of rs650439 genotypes was consistent with the Hardy–Weinberg equilibrium (AA, AT, and TT genotype; 188, 189, and 40, respectively). This distribution is similar to the rs650439 distribution reported for Asian populations in the NCBI database. No significant differences across gender, age, blood pressure, and prevalence of other risk factors among the genotypes were observed (Supplementary Table 1). Consistent with the previous study, we also did not find any difference in IMT among the genotypes in these general subjects (Supplementary Table 1) [14].

**Figure 2:**
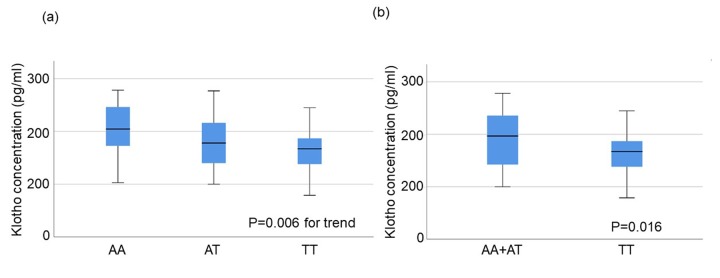
**Plasma klotho concentration in subjects aged 70±1 years stratified by rs650439.** (**a**) Comparison among three groups (AA, AT, TT). The significance of trend in klotho concentration among the groups was determined by Jonckheere-Terpstra trend test. (**b**) Comparison between two groups (AA+AT vs. TT). The significance of difference between two groups was determined by Mann-Whitney U test.

Twenty-nine individuals from each of three groups (AA, AT, TT) were randomly selected and plasma concentrations of klotho protein were measured for each individual. There was no difference in the background characteristics among the genotypes (Table 3). Median klotho concentrations and the interquartile ranges (IQR) were 204.4 (IQR:162.4.-248.7), 177.9 (IQR:138.5-217.4), and 166.9 (IQR:133.8-186.8) (pg/ml) in subjects with AA, AT, and TT genotypes, respectively. A Jonckheere-Terpstra test showed a significant trend of decreased plasma klotho concentration with increased number of T alleles in rs650439 (AA>AT>TT) p=0.006) (Figure 2a). Patients with the TT genotype had significantly lower plasma klotho concentrations than those with AA+AT. (p=0.016) (Figure 2b).

**Table 3:** The characteristics of study subjects in the analysis of plasma klotho concentration.

	All(n=87)		AA(n=29)	AT(n=29)	TT(n=29)	P value		AA+AT(n=58)	P value
male, n(%)	34(39)		10(35)	11(38)	13(45)	p=0.71		21(36)	p=0.44
age, year	70±1		69±1	70±1	70±1	N/A		70±1	N/A
BMI, kg/m^2^	23.2±2.9		23.5±3.0	22.7±3.0	23.5±2.7	p=0.44		23.1±3.0	p=0.56
systolic BP, mmHg	136±32		133±32	132±42	143±17	p=0.38		133±37	p=0.08
diastolic BP, mmHg	79±18		79±18	76±24	81±9	p=0.49		77±21	p=0.07
hypertension, n(%)	39(46)		14(48)	13(46)	12(43)	p=0.92		27(47)	p=0.70
diabetes, n(%)	14(17)		7(24)	4(15)	3(11)	p=0.38		11(20)	p=0.30
dyslipidemia, n(%)	35(42)		10(35)	12(44)	13(46)	p=0.62		22(39)	p=0.53
smoking, n(%)	3(5)		2(7)	0(0)	1(4)	p=0.36		2(3)	p=1.00
drinking, n(%)	21(24)		6(21)	7(24)	8(28)	p=0.83		13(22)	p=0.60
Treatment of hypertension, n(%)	28(33)		11(58)	9(36)	8(35)	p=0.84		20(39)	p=0.72
past history of CVD	5(6)		3(10)	0(0)	2(7)	p=0.26		3(6)	p=0.73

BMI, body mass index; BP, blood pressure; CVD, cerebrovascular diseases

*The significance of differences among three genotypes were determined by ANOVA for continuous variables or chi-square analysis for categorical variables.

†The significance of difference vs. patients with TT were determined by Student’s t-test for continuous variables or Pearson’s Chi-square analysis for categorical variables.

## DISCUSSION

Hypertension is widely recognized as the most important risk factor for stroke [20]. A relationship between *klotho* and hypertension was suggested in a report that *klotho* polymorphisms are correlated with blood pressure levels [21]. In the current investigation, data collected from a population of hypertensive patients in the NOAH study were used to assess the association between a polymorphism in the *klotho* gene and the onset of stroke without the confounding influence of non-hypertensive individuals. The rs650439 genotype was significantly associated with prevalence of stroke in study subjects. In a previous report, a *klotho* functional variant with a missense mutation was associated with an increased incidence of stroke [13]. Previous research also showed that the presence of rs650439 was associated with changes in carotid intimal medial thickness in hypertensive patients [14]. New findings in the current study are consistent with these results in previous studies in demonstrating a significant correlation between *klotho* functional polymorphisms and the onset of cerebrovascular diseases in patients with hypertension.

Circulating klotho concentration is negatively correlated with age in healthy subjects [16]. Plasma klotho concentrations were measured in elderly subjects of the same age (70 years old +/- 1 year) to minimize the influence of age. In this population, a significant correlation between plasma klotho concentration and rs650439 was observed, with a reduction in plasma klotho concentration in the order AA>AT>TT. Interestingly, the recent study showed that hypertensive patients with arterial stiffness had lower circulatory klotho concentration than normal subjects [22]. In addition, recent studies have also suggested that secreted klotho has a unique role distinct from its transmembrane form including the maintenance of baroleflex sensitivity and the protection from memory deficit in mammalian [23,24]. Together, it is conceivable that there is a causable link between rs650439, klotho concentration, and the onset of stroke.

The rs650439 sequence is located in intron 4 of the *klotho* gene, near the end of the exon. It is conceivable that rs650439 is associated with the other functional SNPs of *klotho*, and/or that rs650439 modulates klotho protein metabolism. In a previous haplotype analysis, most SNPs involved in the same haplotype block with rs650439 were located in introns, and the only SNP in a coding region (exon4) is synonymous (Ala to Ala) [14].

To assess how rs650439 genotype affects klotho protein concentration, we sequenced the *klotho* promoter region to identify SNPs associated with rs650439, and successfully detected three SNPs between -527 to -106 in the *klotho* promoter region. Linkage disequilibrium between these three SNPs and rs650439 were analyzed using SNPAlyze but no significant association was detected (data not shown). rs650439 appears to affect the onset of stroke via regulation of klotho plasma concentration, although no SNP associated with rs650439 was detected that could modulate transcriptional activity of the *klotho* gene.

Klotho protein concentration can also be affected by transcriptional regulation of two splicing variants of the *klotho* gene that encode membrane and secreted forms of the klotho protein [7]. The secreted form, encoded by the transcript that consists of exons 1,2, and 3, is a major source of circulating klotho. Thus, polymorphisms in the *klotho* gene that would influence dominance of one of the two splicing variants could also affect circulating klotho concentrations. Further study will be required to investigate whether rs650439 or related polymorphisms of the SNP could influence splicing of the *klotho* gene.

It is well-established that Klotho exerts its function primarily via an interaction with fibroblast growth factor 23 (FGF23) [25]. Interestingly, it was recently reported that high plasma FGF23 concentration is associated with the incidence of coronary disease [26] cardioembolic stroke [27] and stroke [28]. The recent study identified genetic variants associated with circulating FGF23 concentrations [29]. Further investigation will be required to investigate how the *klotho* functional variant would affect the onset of stroke in association with these genetic variants affecting FGF23 concentrations

Some limitations in this study should be noted. First, while an association between the genotype and onset of stroke was observed, the allele frequency of rs640539 was not in Hardy-Weinberg equilibrium. As data in the NOAH study were partly obtained by postal survey of non-ambulatory patients during follow up, potential negative effect of TT genotypes on morbidity and mortality might have increased dropout from the survey, and thus have influenced the analyzed distribution of the genotypes. Thus, the result needs to be validated by replication in different patient cohorts. Second, while rs650439 was correlated with plasma klotho concentrations in the general population, an association between rs650439 and klotho protein concentrations in hypertensive patients that were investigated in the Kaplan-Meier analysis was not addressed. In our previous report and the current analysis in general subjects in SONIC, rs650439 was associated with change in carotid IMT in hypertensive patients but not in general population, suggesting that rs650439 manifests its influence on atherosclerosis only in high-risk population [14]. Further analysis will be required to assess the direct association in rs650439, klotho concentration, and the clinical manifestations of atherosclerosis in hypertensive patients. Third, circulating klotho concentration was assessed only in subjects aged 70 years. Further study will be required to assess the association between rs650439 genotypes and klotho concentration among populations of different ages.

In conclusion, the present study indicates that an allele of *klotho* SNP rs650439 is associated with increased risk of stroke in a hypertensive population. In addition, this genotype was correlated with decreased plasma klotho concentration in the general population. Given the known protective function of *klotho* in cardiovascular disease, these findings suggest a possible link between genetic variation in *klotho*, circulating klotho concentration, and the onset of stroke. Future studies will be required to confirm the importance of the *klotho* SNP in the development of cerebrovascular disease.

## MATERIALS AND METHODS

### Subjects and DNA samples in the analysis of stroke onset

Study subjects were recruited from the out-patient clinic in Osaka University Hospital. All patients participated in the Non-Invasive Atherosclerotic Evaluation in Hypertension (NOAH) study, which was a cohort study for essential hypertension [17–19]. The study design was described in a previous article [19]. Briefly, 813 Japanese subjects were recruited between January 1998 and June 2004, and individuals with malignant diseases and atrial fibrillation were excluded. Blood pressure and BMI were measured during each patient’s first visit. DNA samples were obtained only with written informed consent and the protocol was approved by the Ethical Review Committee of the Osaka University Graduate School of Medicine. DNA was isolated from peripheral blood leukocytes using QIAamp DNA Mini kits (QIAGEN).

### Genotyping of klotho SNP rs650439

Genotypes of rs650439 were determined using the TaqMan SNP genotyping assay (Applied Biosystems Japan, Tokyo, Japan), as previously described [14]. The reaction was carried out following the manufacturer’s protocol, and products were analyzed using ABI PRISM 7900HT (Applied Biosystems Japan, Tokyo, Japan).

### Follow-up evaluation

Clinical follow-up was conducted every year from 2003 to 2009 by telephone, mailed questionnaires, and clinical visits [19], except in cases where stroke occurred prior to 2009. Questionnaires requested information on the occurrence of cardiovascular diseases including stroke, and on the cause of death, if appropriate. Stroke was diagnosed when neurological disturbance was seen for more than 24 hours and cerebral infarction or bleeding was confirmed by computed tomography (CT) or magnetic resonance imaging (MRI).

### Association of rs650439 with plasma klotho protein concentration in 70-year-old patients

To evaluate the influence of rs650439 genotype on plasma klotho concentration, we collected blood samples of 70 ± 1 year-old community-dwelling adults in Japan who participated in the SONIC (Septuagenarians, Octogenarians, Nonagenarians Investigation with Centenarians) study [30–36]. Initially, genotypes of rs650439 were determined using the TaqMan SNP genotyping assay (Applied Biosystems Japan, Tokyo, Japan). Next, 87 individuals were randomly chosen and subsequently divided into AA, AT, and TT genotype groups. Plasma klotho protein concentration was then measured in these patients using human soluble α-klotho ELIZA kits (Immuno-Biological Laboratories Co., Gunma, Japan).

### Statistical analysis

All numerical values were expressed as means ± SD. All statistical tests were two-sided, and a p-value of < 0.05 was considered statistically significant. Differences between means of parameters in groups were tested by the Student’s t-test or the Mann-Whitney U test. The Chi-square test examined intergroup differences of dichotomous variables. The trend among the three groups was assessed using the Jonckheere-Terpstra test. Plasma klotho concentration data were reduced by exclusion of outliers using the 1.5×IQR rule in which data points above the third quantile or below the first quartile are considered outliers. The time-oriented incident rates were analyzed by the Kaplan-Meier method. The Log-rank test examined the differences between the Kaplan-Meier plots. In the multivariate analyses, we tested several models by the stepwise way using combinations of parameters as independent variables for the calculation of both regression coefficients (R-squared) and Akaike’s information criterion (AIC). Among the candidate models, we selected the best-fit model using for each dependent variable. Statistical analyses were performed with JMP 14.0 (SAS Institute Inc., Cary, NC, USA) and SPSS statistics package, version 25.0 (IBM Corporation, Armonk, NY, USA).

## Supplementary Material

Supplementary Table
