# Supplementary material for: A klotho gene single nucleotide polymorphism is associated with the onset of stroke and plasma klotho concentration
Source: Aging (Albany NY). 2018 Dec 31;11(1):104–14. doi: 10.18632/aging.101728 (PMC6339800; doi:10.18632/aging.101728)
Supplement: Supplementary Table [file aging-11-101728-s001.pdf]

SUPPLEMENTARY MATERIAL

Supplementary Table 1. The characteristics of SONIC study.

	All(n＝417)	AA(n=188)	AT(n=189)	TT(n=40)	P value	AA＋AT (n=58)	P value
male, n(%)	204(49.2)	85(45)	96(51)	23(59)	p=0.23	181(48)	p=0.20
age, year	70±1	70±1	70±1	70±1	N/A	70±1	N/A
BMI, kg/m^2	29.3±6.5	22.9±3.3	22.8±3.1	23.5±2.5	p=0.44	22.8±3.2	p=0.20
systolic BP, mmHg	136±26	136±22	136±37	134±36	p=0.89	136±25	p=0.64
diastolic BP, mmHg	78±15	79±13	78±16	76±20	p=0.59	78±14	p=0.40
hypertension, n(%)	266(64)	121(65)	118(63)	27(68)	p=0.86	239(64)	p=0.65
diabetes, n(%)	65(16)	26(14)	30(16)	9(23)	p=0.38	56(15)	p=0.21
dyslipidemia, n(%)	184(44)	92(49)	73(39)	19(48)	p=0.11	165(44)	p=0.68
smoking, n(%)	124(30)	51(28)	63(34)	10(26)	p=0.36	114(31)	p=0.54
drinking, n(%)	142(35)	66(36)	65(35)	11(28)	p=0.52	131(36)	p=0.44
Treatment of hypertension, n(%)	149(72)	60(65)	77(78)	12(80)	p=0.12	137(72)	p=0.49
past history of CVD	9(4)	5(5)	3(3)	1(3)	p=0.72	8(4)	p=0.88
mean IMT, mm(IQR)	0.76(0.68-0.88)	0.75(0.68-0.88)	0.78(0.70-0.88)	0.80(0.73-0.85)	p=0.41	0.75(0.68-0.88)	p=0.44

BMI, body mass index; BP, blood pressure; CVD, chronic vascular disease; IMT, intima-media thickness; IQR, interquartile range;
*The significance of differences among three genotypes was determined by ANOVA or Kruskal-Wallis test for continuous variables and chi-square analysis or Fisher's exact test for categorical variables.
